# World health system performance revisited: the impact of varying the relative importance of health system goals

**DOI:** 10.1186/1472-6963-4-19

**Published:** 2004-07-22

**Authors:** Jeremy A Lauer, CA Knox Lovell, Christopher JL Murray, David B Evans

**Affiliations:** 1 Evidence and Information for Policy, World Health Organization, Geneva, Switzerland; 2 Department of Economics, University of Georgia, Athens, GA 30602, USA

**Keywords:** health care provision, weighting of indicators

## Abstract

**Background:**

In 2002, the World Health Organization published a health system performance ranking for 191 member countries. The ranking was based on five indicators, with fixed weights common to all countries.

**Methods:**

We investigate the feasibility and desirability of using mathematical programming techniques that allow weights to vary across countries to reflect their varying circumstances and objectives.

**Results:**

By global distributional measures, scores and ranks are found to be not very sensitive to changes in weights, although differences can be large for individual countries.

**Conclusions:**

Building the flexibility of variable weights into calculation of the performance index is a useful way to respond to the debates and criticisms appearing since publication of the ranking.

## Background

The World Health Organization recently published a performance ranking of the health systems of its 191 member countries, and intends to update it at regular intervals [1-4]. It was based on a framework outlining a set of social goals to which health systems should contribute [5]. It was argued that systems should contribute to improving population health, be responsive to the people they serve and be financed fairly. Five outcome indicators were defined – the level of population health, inequalities in health, the level of responsiveness, inequalities in responsiveness and fairness in financial contributions. Estimates of attainment on these five indicators were made for the 191 countries that were members of WHO at that time, and a composite (overall) attainment indicator was constructed for each country as a weighted average of attainment on the five individual outcome indicators.

Publication of the analytical framework and the resulting ranking provoked considerable comment, and a variety of issues concerning the methodology and country positions in the ranking have been raised. A central component of the methodology for measuring overall attainment was the use of fixed weights, common to all countries, to aggregate the five indicators. This feature has been controversial, with some arguing that people in different cultural and social settings value individual health system goals in different ways [6-13]. The fixed weights had the virtue of being based on expert opinion, having been derived from the valuations of 1,007 respondents – largely health system professionals – to a WHO survey [14]. However the weights were common to all countries, regardless of their development status and cultural traditions.

In this paper we examine the sensitivity of the attainment scores to alternative weighting schemes that allow weights to vary across countries. These country-specific weights may reveal varying objectives of policy makers or constraints under which they operate. Melyn and Moesen [15] have referred to such weights as 'benefit of the doubt' weights.

## Methods

WHO used fixed weights (0.25, 0.25, 0.125, 0.125, 0.25) to aggregate five health system outputs (respectively, the level of population health, inequality in the distribution of health, the level of health system responsiveness, inequality in the distribution of responsiveness, and fairness in financial contributions) into a scalar health system attainment index. Overall attainment ranged from 35.7 (Sierra Leone) to 93.4 (Japan) on a [0–100] scale.

We propose here an analytical framework that reduces to the WHO fixed-weight methodology as a special case, but that allows varying degrees of freedom for weights to be defined that – in the sense of Melyn and Moesen – implicitly take into account individual country circumstances. For shorthand, we say countries "choose" such weights, which in reality are determined as solution values of a linear program. If the linear program is a fair representation of the objective function of and constraints faced by decision makers, the weights are indeed "chosen", but even if this condition is not necessarily met, the resulting weights may still be of interest.

The extent of freedom to choose weights in a linear program can be set by the analyst. The analyst can enforce fixed weights common to all countries, allow countries complete freedom to choose their own weights, or adopt a middle ground in which countries are granted limited freedom to choose weights within bounds thought to be sensible by experts.

A generic statement of the performance evaluation problem in primal-dual linear programming format is:


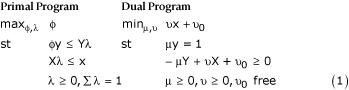


In these programs y is a country's output vector, x is its input vector, Y is the sample output matrix and X is the sample input matrix. In the present context y is (5 × 1), Y is (5 × 191), x is (n × 1) and X is (n × 191), with n to be specified below.

The primal program seeks the maximum radial expansion of a country's outputs, provided that it not exceed the standards established by a convex combination (λ ≥ 0, ∑λ = 1) of best-practice countries in the sample. The optimal value of φ provides a distance measure (i.e. an indication of how far a country has to go) to match best practice as observed in the sample. Since φ ≥ 1, the attainment of a country is evaluated as φ^-1 ^≤ 1. Best practice countries have φ^-1 ^= 1, other countries have φ^-1 ^< 1, so φ provides a basis for acomplete ranking of countries on their relative ability to deliver five health system outputs. The dual program seeks a set of nonnegative weights μ,υ attached to a country's outputs and inputs that maximize its attainment. Each country's output weights are normalized by μy = 1, but each is free to select its own set of nonnegative weights.

In constructing the overall attainment index, WHO identified five output indicators and no inputs, preferring to treat each country's health system as a "health output management unit". Consequently each country's input vector is represented as a scalar with unit value. Under these circumstances the performance evaluation problem simplifies to:


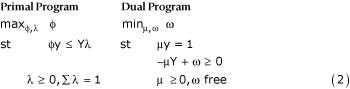


where ω = υ + υ_0_. The modified primal program seeks the maximum feasible radial expansion of a country's outputs consistent with best practice observed in the sample. The modified dual program seeks a set of nonnegative weights μ for a country's outputs that put it in the best light. A country can be expected to assign relatively large weights to those outputs at which it excels relative to best practice, and relatively low, possibly zero, weights to those outputs at which it lags behind best practice, subject to the normalization μy = 1. (See Annex for a graphical explanation.)

By complementary slackness, μ_m_(Y_m_λ - φy_m_) = 0, m = 1, ..., M, so slack in any element of a country's projected output vector φy implies that the country assigns a zero weight to that output. Since it is unreasonable to allow a country to assign a zero weight to any output deemed sufficiently important to have been included in the WHO performance evaluation exercise, it is desirable to restrict weights in some way. This can be accomplished most easily by appending constraints to the dual side of (2) of the form:

γ_m _≥ μ_m_y_m_/μy ≥ β_m_, m = 1, ..., M.     (3)

Restrictions (3) place lower and upper bounds on the relative importance of each output (as measured by μ_m_) in total output.

Implementation of the weight-restricted linear program requires specifying the 2M = 10 parameters γ_m_, β_m_. One procedure is to ignore the upper bounds γ_m _and set the lower bounds β_m _> 0. This eliminates the possibility of a country assigning zero weights to those outputs at which it lags behind best practice. A less arbitrary procedure is to follow Takamura and Tone [16] by adapting Saaty's Analytical Hierarchy Process (AHP) [17]. This procedure exploits expert judgment, that could be provided for example by the above-mentioned survey of health system professionals, to set lower bounds β_m _> 0 and upper bounds 1 > γ_m_. Although these bounds are common to all countries, they allow countries limited freedom to select weights appropriate to their circumstances.

## Results

The fixed weights used by WHO)) [4] to aggregate the five health system indicators gave countries no freedom to choose weights appropriate to their circumstances. We compare the WHO attainment index with three alternative indexes allowing countries varying degrees of freedom to choose weights. The first index is based on the solution to program (2), without weight restrictions, thereby allowing complete freedom to choose. The second index is based on (2), with lower bounds in (3) of β_m _= 0.10 on all weights, allowing substantial freedom to choose. The third index is based on (2), with lower and upper bounds in (3) set by a modified AHP procedure.

In the modified procedure, "expert opinion" was taken to mean the average values of weights arising from population-representative country surveys, each of which included a module on health system goals [9]. Respondents were queried about their individual preferences on the five stated health system goals in a total of 51 countries, in some of which multiple surveys were performed, and country means were calculated on the basis of these individual responses [18]. The survey methods, reliability, validity, representativeness, sample size and respondent characteristics are extensively described in Ustün et al. [9], and are also reported in summary form in Sadana et al. [19], Mathers et al. [20], Mathers et al. [21] and Sadana et al. [22]. The survey instruments are available at .

For each output, the lower bound for calculation of the third index was taken as the minimum of the country average weights, and the upper bound the maximum [23]. Country mean weights and survey types, as well as survey wide maximum and minimum weights are shown in Table 1. The lower bounds are accordingly β_m _= (0.19, 0.17, 0.12, 0.11, 0.22) and the upper bounds γ_m _= (0.29, 0.25, 0.18, 0.17, 0.30). This specification allows limited freedom to choose. We refer to the four indexes as WHO, LP1, LP2 and LP3, respectively.

Summary statistics of the four attainment indexes appear in Table 2. The three LP distributions have higher means than the WHO distribution, and two of them have lower dispersion. However increasing restrictions on freedom to choose reduce the mean, and increase the dispersion, of the LP attainment indexes toward the mean and dispersion of the WHO attainment index.

Rank correlations between pairs of attainment rankings appear in Table 3. Despite the distributional changes due to freedom to choose, rank correlations are positive, high and statistically significant. The lowest correlations involve LP1, the index allowing complete freedom to choose. With the exception of LP1, there is strong agreement about the identity of countries in the top and bottom quartiles of the distribution. Japan is ranked #1 and Sierra Leone is ranked #191 on all four indexes.

**Figure 1:**
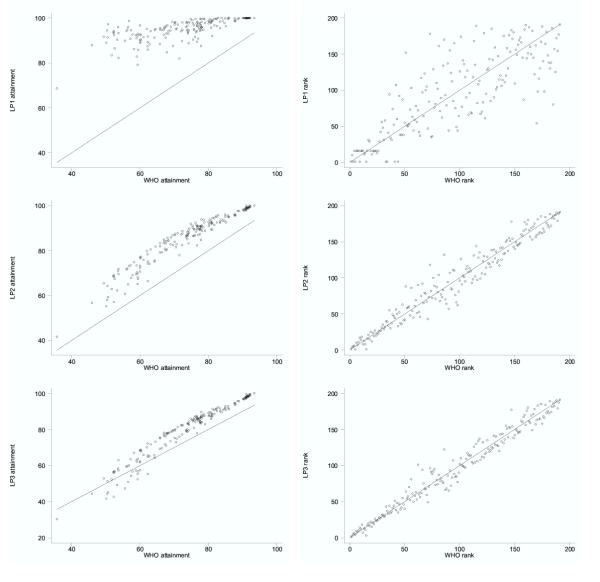
Graphical array showing LP attainment scores vs WHO attainment scores (left column) and LP ranks vs WHO ranks (right column). From top to bottom, WHO vs LP1, LP2, and LP3, respectively.

Figure 1 shows plots of WHO attainment scores and rankings versus the three LP attainment scores and rankings. The convergence of the distributions of the LP attainment scores and rankings to the WHO scores and rankings is apparent.

Results based on LP1 are unattractive. Over one-third of countries (70 of 191) assign a zero weight to four of the five indicators, and the vast majority of countries assign a weight in excess of 0.9 to either responsiveness distribution or fairness in financial contributions. This means they assign a low or zero weight to population health, the defining goal of the health system, which does not have face validity. In LP1, only Japan, Luxembourg and the United States assign positive weights to all five indicators. Consequently the attainment index is tightly distributed about a very high mean value. The ability to discriminate is sacrificed to freedom to choose, with 75% of countries receiving attainment indexes of 99 or above.

Results from LP2 are somewhat more attractive. Nevertheless, when weights are bounded below by 0.1, over three-quarters (147 of 191) of countries assign the minimum weight to four of the five indicators and a 0.6 = 1 - (4 × 0.1) weight to either responsiveness distribution or fairness in financial contributions. The attainment scores are again compressed about a high mean value, and the ability to discriminate is not much improved, with 75% of countries receiving attainment scores of 93 or above. However despite this dramatic compression, the LP2 ranking is globally very similar to the WHO ranking. Eighteen of the countries ranked in the top 20 by WHO appear in the LP2 top 20, and 14 of the countries ranked in the bottom 20 by WHO appear in the LP2 bottom 20.

Not surprisingly, the distribution of the attainment scores for LP3 looks even more similar to the distribution of the WHO scores, and has a similar mean and standard deviation. Rank correlation is very high, with only one country falling out of the WHO top 20 and only five countries rising out of the WHO bottom 20. Nonetheless, limited freedom to choose has an important impact on individual countries. The USA, given an ability to emphasize the importance of responsiveness level and responsiveness inequality, and to de-emphasize the importance of health level and inequality in the distribution of health, improves its ranking from #15 to #3. Australia improves from #12 to #7 for similar reasons. Italy, on the other hand, falls from #11 to #18, primarily as a result of the movement of other countries. In LP3, the largest positive changes in ranking are for Mauritius (+25) and Saint Vincent (+22), while the largest negative changes in ranking are for Kazakhstan (-39) and Albania (-36).

## Discussion

We began by questioning the appropriateness of the fixed weight approach to aggregating indicators adopted by WHO, on the grounds that fixed weights deny countries at varying stages of development the freedom to choose. We then proposed a sequence of linear programming models that allowed countries varying freedom to choose the weights assigned to their indicators. LP1 allows complete freedom to choose, and generates weights we consider unacceptable, particularly because so many countries give a zero weight to improving health. LP2 allows considerable freedom to choose, but generates many country-specific weights falling outside the range of the within-country means used as bounds in LP3. Clearly, the validity of a procedure that routinely assigns weights out of the range of representative cross-population preferences should be questioned, even without a sophisticated theory of empirical ethics or democratic choice.

LP3 applies the AHP procedure to set plausible bounds on weights, and allows limited freedom to choose. It generates a very similar distribution of the attainment index, and a very high linear rank correlation with the WHO ranking. Despite these similarities, we find the LP3 approach intuitively appealing, and are encouraged by its global concordance with the WHO index. However it is fair to ask: if LP3 and WHO generate such similar rankings, why bother? What value does LP3 add? Indeed, individual countries may come to diametrically opposed conclusions about the benefits of LP3 or WHO on the basis of their implied rank changes (e.g. Mauritius vs. Kazakhstan).

Howbeit, our first response to the question "why bother" focuses on the distribution of the LP3 weights in comparison to the WHO weights. The WHO weight on responsiveness inequality was 0.125. But the LP3 upper bound of 0.17 is binding on 182 countries, which implicitly want a higher weight on this indicator. The WHO weight on fairness in financial contributions was 0.25. But the LP3 upper bound of 0.30 is binding on 167 countries that want a higher weight. At the other end, the WHO weight on health level is 0.25. But the LP3 lower bound of 0.19 is binding on 170 countries that want a lower weight. The WHO weight on responsiveness level is 0.125. But the LP3 lower bound of 0.12 is binding on 103 countries that want a lower weight. It appears that a majority of countries at all stages of development may implicitly assign greater importance to indicators of health distribution, and less importance to indicators of health level, than the experts whose judgments formed the basis of the original WHO weights.

Our second response is more general. By allowing countries limited freedom to choose their weights, LP3 takes a small but nevertheless significant step toward respecting their varying circumstances. While the narrower the bounds on weights, the smaller the step, even the limited freedom embodied in LP3 makes an important difference to some countries.

## Conclusions

Building in the flexibility of varying weights might be a useful way for WHO to respond to the debates and criticisms appearing since publication of the ranking. We conclude by speculating that a variant of LP3 incorporating information regarding which weights are binding, and in which direction, might yield even greater benefits in terms of respecting individual circumstances.

## Competing interests

JAL, CJLM and DBE were part of the team at WHO that developed the methods for the world health system performance rankings published by the Organization in 2000.

## Abbreviations

AHP (Analytical Hierarchy Process)

DEA (Data envelopment analysis)

LP (Linear program)

LP1 (Linear Program 1)

LP2 (Linear Program 2)

LP3 (Linear Program 3)

WHO (World Health Organization)

## Authors' contributions

JAL developed methods inspired by the benefit-of-the-doubt concept and drafted an earlier version of the manuscript. CAKL adapted and applied the DEA methods described here to health system performance and drafted the initial version of the current manuscript. CJLM conceived the methods for measuring health system performance. DBE coordinated the research at WHO. All authors revised and approved the manuscript.

## Pre-publication history

The pre-publication history for this paper can be accessed here:



## Supplementary Material

Additional File 1 Annex.Graphical interpretation of DEA and conceptual links with value theory.doc Click here for file

## Figures and Tables

**Table 1:** Country means and survey types.

	Mean survey weight					
Survey country	Health level	Health distribution	Responsiveness level	Responsiveness distribution	Fair financing	Survey type
Argentina	0.26	0.25	0.13	0.13	0.24	brief^a^
Australia	0.23	0.21	0.15	0.14	0.27	postal^b^
Austria	0.23	0.20	0.15	0.14	0.28	postal
Bahrain	0.26	0.17	0.17	0.13	0.27	brief
Belgium	0.24	0.25	0.13	0.14	0.25	brief
Bulgaria	0.25	0.22	0.13	0.13	0.27	brief
Canada	0.25	0.17	0.16	0.13	0.29	brief
Chile	0.20	0.21	0.15	0.15	0.29	brief
China	0.27	0.21	0.15	0.12	0.25	postal
Costa Rica	0.28	0.25	0.12	0.12	0.22	brief
Croatia	0.25	0.24	0.13	0.13	0.25	brief
Cyprus	0.25	0.23	0.14	0.13	0.25	postal
Czech Republic	0.29	0.22	0.14	0.12	0.24	brief, postal
Denmark	0.26	0.21	0.15	0.12	0.25	postal
Egypt	0.26	0.22	0.14	0.12	0.26	postal
Estonia	0.27	0.22	0.14	0.11	0.25	brief
Finland	0.25	0.21	0.14	0.12	0.27	brief, postal
France	0.24	0.23	0.14	0.13	0.26	brief, postal
Germany	0.24	0.22	0.13	0.14	0.28	brief
Greece	0.25	0.21	0.15	0.13	0.26	postal
Hungary	0.24	0.20	0.16	0.13	0.27	postal
Iceland	0.26	0.20	0.14	0.13	0.26	brief
Indonesia	0.26	0.22	0.15	0.13	0.24	brief
Ireland	0.24	0.25	0.13	0.13	0.26	brief
Italy	0.24	0.23	0.14	0.14	0.25	brief
Jordan	0.26	0.18	0.16	0.12	0.27	brief
Kyrgyzstan	0.25	0.22	0.13	0.13	0.26	postal
Latvia	0.24	0.19	0.15	0.12	0.30	brief
Lithuania	0.24	0.23	0.15	0.13	0.25	postal
Luxembourg	0.25	0.23	0.13	0.13	0.26	telephone^c^
Malta	0.25	0.23	0.13	0.14	0.24	brief
Morocco	0.19	0.18	0.17	0.17	0.29	brief
Netherlands	0.24	0.18	0.17	0.14	0.28	brief, postal
New Zealand	0.24	0.22	0.15	0.13	0.26	brief
Oman	0.25	0.18	0.18	0.14	0.25	brief
Poland	0.24	0.22	0.14	0.12	0.27	brief
Portugal	0.24	0.25	0.13	0.13	0.25	brief
Republic of Korea	0.28	0.21	0.14	0.12	0.25	brief
Romania	0.25	0.22	0.14	0.13	0.26	brief
Russian Federation	0.25	0.17	0.17	0.12	0.29	brief
Spain	0.26	0.24	0.13	0.13	0.24	brief
Sweden	0.23	0.20	0.15	0.14	0.28	brief
Switzerland	0.22	0.21	0.15	0.15	0.27	postal
Thailand	0.21	0.19	0.16	0.16	0.28	brief
Trinidad and Tobago	0.24	0.22	0.14	0.13	0.26	brief
Turkey	0.24	0.23	0.13	0.13	0.27	postal
Ukraine	0.26	0.22	0.14	0.12	0.26	brief
United Arab Emirates	0.23	0.17	0.18	0.14	0.28	brief
United Kingdom	0.26	0.22	0.14	0.12	0.26	postal
United States of America	0.24	0.20	0.15	0.16	0.25	postal
Venezuela	0.28	0.23	0.13	0.11	0.25	brief
Sample minimum	0.19	0.17	0.12	0.11	0.22	
Sample maximum	0.29	0.25	0.18	0.17	0.30	

**^a^**brief = brief face-to-face survey (Üstün et al. 2001). **^b^**postal = postal survey (Üstün et al. 2001). **^c ^**telephone = computer-assisted telephone survey (Üstün et al. 2001).

**Table 2:** Summary Statistics

	WHO	LP1	LP2	LP3
Mean (%)	73.30	95.18	85.15	79.28
Standard deviation (%)	12.34	4.79	11.34	14.98
Interquartile range (%):				
First quartile	63.64	92.99	78.12	69.42
Second quartile	75.39	96.00	87.91	83.37
Third quartile	81.65	99.05	93.25	89.83
Fourth quartile	93.45	100.00	100.00	100.00

**Table 3:** Rank Correlations.

	WHO	LP1	LP2	LP3
WHO	1.00	0.71	0.96	0.97
LP1		1.00	0.75	0.69
LP2			1.00	0.99
LP3				1.00
