# Supplementary material for: World health system performance revisited: the impact of varying the relative importance of health system goals
Source: BMC Health Serv Res. 2004 Jul 22;4:19. doi: 10.1186/1472-6963-4-19 (PMC506780; doi:10.1186/1472-6963-4-19)
Supplement: Additional File 1 — Annex.Graphical interpretation of DEA and conceptual links with value theory.doc [file 1472-6963-4-19-S1.doc]

# Annex: Graphical interpretation of DEA and conceptual links with value theory.

health indicator #2

A

B

F”

F’ C

**F**

D

E G”

G'

**G**

O health

indicator #1

## Annex Figure: Graphical representation of DEA vs WHO problem.

The programming technique used in the body of the paper is a variant of Data Envelopment Analysis (DEA), originally developed by Charnes et al.[1]. In this Annex, the DEA programming technique is explained graphically. The five health system indicators we analysed in the body of the paper are reduced to two for the sake of the two-dimensional diagram (Annex Figure).

In DEA terms, countries A, B, C, D, E are best-practice, or efficient, countries because each is undominated in the sense that their output points lie on the convex hull of the data points. Note that each country provides different mixes of health system outputs. In DEA, the convex hull (in two dimensions, piece-wise linear envelope) formed by the output points is the health system production possibilities frontier, and is assumed to be conditioned on extant technology.

Countries F and G are inefficient, since their output points are located on the interior of the production possibilities set. In DEA terms, the efficiency of countries F and G are measured radially as OF/OF’ and OG/OG’. The research question was to compare DEA efficiencies with the fixed-weight WHO efficiency index so as to understand the sensitivity of the WHO index to different assumptions about weights. Under all assumptions, the rank correlation was high. Restricting consideration to only non-zero weights, other distributional measures support the conclusion of limited sensitivity of the rank index, with nevertheless, important local differences (see Results).

In measuring efficiency as OF/OF’ and OG/OG’ in DEA, the countries’ outputs geometrically determine the weights used in measuring their efficiencies. To see this, note that the output of country F is projected to a point F' on the facet BC, and the implicit weight ratio is therefore the slope of the facet BC. An assumption of DEA is that this slope provides a "plausible" weight ratio. However, note that country G is relatively good at providing health indicator #1, and not so good at providing health indicator #2. In unconstrained DEA, its output point is projected to the vertical facet extending down from E, and the implicit weight it attaches to health indicator #2 is zero. Under weak assumptions about the importance of indicator #2, this is unlikely to be plausible.

Weight restrictions in DEA work like restrictions on the slopes of facets. Slopes of facets express the weight ratio. A simple restriction might impose weights of at least 0.10, thereby eliminating the possibility of vertical and horizontal facets. With such a restriction, the vertical facet extending south from E would now extend slightly southeast from E, and the performance of country G as measured with respect to this facet of the convex hull would be slightly worse, since the segment GG’ with this restriction is longer than the segment GG’ without the restriction. In other words, country G is no longer allowed to choose its most preferred weights freely. The same restriction would force the facet extending west from A to extend slightly northwest. Restrictions of this type ensure non-zero weights. Other restrictions might impose upper limits and would have the effect of limiting slopes of the interior facets AB, CD, DE.

To see the relationship of the DEA problem with the construction of the WHO index, suppose health indicators #1 and #2 each receive a weight of 0.5 in the WHO measurement problem. Under this assumption, the WHO weight ratio is 1. This weight ratio is represented graphically as the red line in the diagram with a slope of -1, where the negative sign expresses the convexity condition. Essentially, in the WHO problem, the purely technical and comparative performance evaluation exercise (based on output maximization) is transformed into a value-driven performance evaluation exercise (based on revenue maximization). In other words, in the WHO problem, the notion of “relative prices” is substituted for the DEA concept “weight ratio.” Note that F is a little less efficient under WHO than under LP1 (OF/OF”), and G is a lot less efficient under WHO than under LP1 (OG/OG”).

Note that the WHO problem implies relative prices of 0.5 for both goods. The WHO index published in the *World Health Report 2000* used relative prices 0.25, 0.25, 0.125, 0.125, 0.25 for the different health system components. It is clear that the constraint that relative prices (or weights) sum to 1 simply imposes a specific normalization. It has no other significance, since it can be seen from the diagram that the results of the maximization problem are independent of any particular choice of units.

In the two-dimensional WHO problem, the production frontier is a straight line with slope of -1, and the production possibilities set is the triangle bounded by the relative price line and the axes. In other words, since relative prices (weights) are taken as given for all countries, the WHO index can be said to involve stronger assumptions. In DEA, countries choose their own weights (relative prices), subject to optional exogenous constraints.

In terms of value theory, this distinction can be rephrased as a statement about whether health systems are price takers or price setters with respect to the five health system outputs. However, since in most formulations of the DEA problem, some constraints on weights are necessary in order to obtain a plausible solution, the actual case seems intermediate: countries are neither price takers or price setters, but possibly should be accorded limited freedom to determine health-system-output prices/weights.

## Reference

1. Charnes A, Cooper WW, Rhodes E: **Measuring the efficiency of decision-making units.** *European Journal of Operational Research* 1978, **2:** 429-444.
